# Heterologous Expression of a Membrane-Spanning Auxin Importer: Implications for Functional Analyses of Auxin Transporters

**DOI:** 10.1155/2009/848145

**Published:** 2009-06-18

**Authors:** David John Carrier, Norliza Tendot Abu Bakar, Karen Lawler, James Matthew Dorrian, Ameena Haider, Malcolm John Bennett, Ian Derek Kerr

**Affiliations:** ^1^School of Biomedical Sciences, University of Nottingham, Queen's Medical Centre, Nottingham NG7 2UH, UK; ^2^School of Biosciences, University of Nottingham, Sutton Bonington Campus, Leics LE12 5RD, UK

## Abstract

Biochemical studies of plant auxin transporters in vivo are made difficult by the presence of multiple auxin transporters and auxin-interacting proteins. Furthermore, the expression level of most such transporters in plants is likely to be too low for purification and downstream functional analysis. Heterologous expression systems should address both of these issues. We have examined a number of such systems for their efficiency in expressing AUX1 from *Arabidopsis thaliana*. We find that a eukaryotic system based upon infection of insect cells with recombinant baculovirus provides a high level, easily scalable expression system capable of delivering a functional assay for AUX1. Furthermore, a transient transfection system in mammalian cells enables localization of AUX1 and AUX1-mediated transport of auxin to be investigated. In contrast, we were unable to utilise *P. pastoris* or *L. lactis* expression systems to reliably express AUX1.

## 1. Introduction

**Figure 1:**
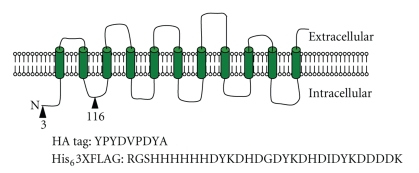
Epitope-tagged forms of AUX1. Diagrammatic representation of AUX1 constructs. The predicted membrane topology of AUX1 is shown with TM helices represented as cylinders. The epitope sequences for the HIS_6_3xFLAG and HA tags are shown with the sites of insertion represented as triangles, with the specific residue number for the insertion site below.

Auxin (indole-3-acetic acid) and several related compounds are key hormones in plants and have a multitude of effects on plant physiology, regulating, amongst other process, tropic responses to light and gravity, organogenesis, and senescence. The polarised transport of auxin into and out of cells is essential to control cellular auxin levels and the generation and maintenance of auxin gradients required for these processes. The AUXIN RESISTANT 1 (AUX1) gene encodes an auxin influx carrier belonging to the AUX/LAX family of auxin influx transporters [1]. Loss of AUX1 function results in reductions in growth and response to gravity [2]. The AUX1 protein comprises a polypeptide of 485 amino acids, with a predicted molecular mass of 54 kDa. The protein is localised to the plasma membrane, with a predicted topology of 11 transmembrane helices, a cytoplasmic N-terminus, and apoplastic C-terminus [3] (Figure 1). Its transport substrate/ligand (auxin, indole-3-acetic acid) is a weak organic acid (pKa of 4.8), structurally similar to the amino acid tryptophan. AUX1 is proposed to function as a proton:auxin symporter since the protein (and its sequence homologues LAX1-3) shares a high degree of sequence identity with the amino acid auxin permease (AAAP) family of transporters [4]. Detailed biochemical characterization of AUX1 and other auxin transporters is critical to understand their contribution to plant development [5].

Several constraints prevent the characterization of the biochemistry of auxin transporters in plants. Firstly, a large number of additional auxin interacting proteins exist (both in cellular membranes as well as intracellularly), including auxin receptors, influx and efflux transporter proteins. Secondly, the expression level of most membrane proteins is relatively low in their natural membrane. For these reasons we attempted to express the AUX1 auxin importer in several heterologous systems. Of these, some are compatible with high level expression, essential for longer term strategies aimed at purification and reconstitution. 

When selecting a system for heterologous protein expression it is important to consider the capabilities of the system which best suit the downstream applications. Among the important considerations are time investment, cost, fidelity of posttranslational modification, compatibility with functional assay, and expression level scalability. For functional studies a system that has similar posttranslational modification machinery would be highly desirable for conservation of function (be that binding of ligand, or transport per se), whereas for purification a system that lends itself to the production of large quantities (mg of protein) is more desirable. We describe the approaches and our experiences with four heterologous expression systems for AUX1. One of these (*Lactococcus lactis*) is a prokaryotic expression system, whereas the other three (*Pichia pastoris*), baculovirus infected insect cells, and transfected mammalian cells are eukaryotic. Of the four systems two enabled us to pursue functional analysis of AUX1 and one will be able to support further studies on the purified and reconstituted protein.

## 2. Materials and Methods

### 2.1. DNA Constructs

AUX1 cDNAs encoding appropriate epitope tags (Figure 1) were generated: an N-terminal haemagluttinin tagged AUX1 construct (N-HA-AUX1) and two constructs with different locations of an insertion comprising a hexahistidine stretch followed by three repeats of the FLAG epitope (N-His_6_-3xFLAG-AUX1 and L2-His_6_-3xFLAG-AUX1). For His_6_3XFLAG cDNAs the template DNAs were two pBluescript derived vectors pSK_AUX1_NYFP and pSK_AUX1_L2YFP described previously [3]. The YFP gene in each vector was removed by Asp718 digestion and replaced by insertion of an Asp718 digested double stranded oligonucleotide (top strand primer sequence: GGGGTACCCACCATCATCATCATCATATCGACTACAAAGACCATGACGGTGATTATAAA GATCATGATATCGATTACAAGGATGACGATGACAAGGGTACCGG). HA-AUX1 cDNA was generated by RT-PCR from *Arabidopsis thaliana* HA-AUX1 transgenic plants [3]. Briefly, RNA was extracted from 7 day old (At) seedlings using a Qiagen RNeasy kit following the manufacturer's instructions, and 1 *μ*g of this was reverse transcribed (2 hours, 37°C) using Superscript II (Invitrogen). The reaction was heat inactivated (70°C for 10 minutes), and the resultant cDNA was amplified by PCR with primers (5′ GGGAATTCTCTAATAGAAAACATCTA and 5′ GGACTAGTTCAAAGACGGTGGTGTAAAGCGGA). Tagged cDNAs were then shuttled into expression vectors (pcDNA3.1(+), pPICZB, pNZ8048, and pFastBac1, all from Invitrogen, except pNZ8048 [6]) using restriction digest and ligation. All plasmids were fully sequenced to ensure in-frame insertion of any epitope tags.

### 2.2. Expression of Epitope-Tagged AUX1 in Mammalian Cells

HEK293T [7] or U2OS [8] cell monolayers were propagated in complete high glucose DMEM (4.5 g/L glucose; GIBCO) supplemented with 10% (v/v) heat-inactivated foetal calf serum (FCS; GIBCO), and 50 units/mL penicillin/streptomycin (GIBCO) at 37°C, 5% CO_2_ and maintained by passaging when approximately 80% confluent. For transfection, cells were seeded at densities of either 400 000 per well (HEK293T) or 100 000 cell per well (U2OS) in a 6-well dish (Falcon) 27 hours before transfection. U2OS cells were transfected with Fugene (Roche) according to the manufacturer's instructions, whereas polyethyleneimine (PEI) was used to transfer DNA into HEK293T cells [9]. For PEI transfection, media was replaced with a lower serum percentage media (2% v/v) 3 hours prior to transfection. At the time of transfection 10 mM linear PEI (Polysciences, Inc.), pH 7, was added to plasmid DNA containing 5% w/v glucose (4–8 *μ*g of DNA per well) to achieve a nitrogen : phosphorus molar ratio of 8 : 1 (the N : P ratio refers to PEI-derived nitrogen : DNA-derived phosphorus [9]). Following brief mixing, this was added dropwise to the cell monolayers. Twenty-four hours posttransfection the media was removed and replaced by 10% v/v serum-containing media supplemented with 2 mM butyric acid. Cells were harvested 48–88 hours posttransfection by repeat pipetting into ice cold phosphate buffered saline (PBS) supplemented with 2 mM EDTA and centrifuged at 300 g for 10 minutes. Cell pellets were resuspended in 250 *μ*L PBS containing protease inhibitors (Complete EDTA-free Protease Inhibitor, Roche) and lysed by 3 × 10 seconds bursts of sonication.

### 2.3. Expression of Epitope-Tagged AUX1 in Sf9 Insect Cells

*Spodoptera frugiperda* (Sf9) cells were grown as orbital cultures at 27-28°C in InsectXpress medium (Lonza) supplemented with 10% v/v foetal calf serum and 50 units/mL penicillin and streptomycin. AUX1 was expressed in Sf9 cells following infection with recombinant baculovirus. Recombinant bacmid DNA was constructed using the Bac-2-Bac system (Invitrogen), following the manufacturer's instructions. After PCR screening of the bacmid DNA to ensure correct insertion of AUX1 cDNA, recombinant virus was generated by Cellfectin-mediated transfection of Sf9 cell monolayers. Baculovirus was amplified and titred using standard methodologies [10]. AUX1 expression was induced by infecting suspension cultures of Sf9 cells at 2.0 × 10^6^/mL at varying multiplicities of infection (MOI), and cells were incubated for 24–96 hours after infection. Cells were harvested by centrifugation (500 g, 5 minutes at 4°C), resuspended in 10 times the pellet volume in 10 mM Tris pH7.4, 250 mM sucrose, 0.2 mM CaCl_2_ with protease inhibitors (as above) and passed twice through a pressure disruptor (Constant Systems) at 5000 psi to lyse.

### 2.4. Expression of Epitope-Tagged AUX1 in Lactococcus lactis

Epitope-tagged AUX1 cDNAs were inserted in the pNZ8048 vector [6] via the *Nco*1 and *Spe*1 restriction sites. Following electroporation of the recombinant plasmids into electrocompetent *L. lactis* NZ9000 cells, chloramphenicol resistant colonies were picked from selective plates and grown at 30°C in M17 medium (Oxoid) supplemented with 0.5% w/v glucose and 5 *μ*g/mL chloramphenicol. Cultures were grown to an A_600_ of 0.6 then induced by adding culture media supernatant from the nisin-producing strain NZ9700 grown to an OD_600_ of 0.9 at a range of dilutions between 1 : 250 and 1 : 20, 000 v/v. Cells were harvested at 1 and 2 hours postinduction by centrifugation (4000 g, 15 minutes at 4°C), washed and resuspended in ice cold 100 mM potassium phosphate buffer, pH 7 with protease inhibitors (as above) and passed through a pressure disruptor at 20 000 psi to lyse.

### 2.5. Expression of Epitope-Tagged AUX1 in Pichia pastoris

Expression of epitope-tagged AUX1 was performed using the EasySelect *Pichia* Expression Kit (Invitrogen) following the manufacturer's instructions. In brief, epitope-tagged AUX1 cDNAs were inserted in the pPICZB vector and zeocin resistant colonies picked from selective plates (low salt LB agar with 25 *μ*g/mL zeocin). Electrocompetent *P. pastoris* KM71H cells were transformed with linear pPICZB_AUX1 constructs by lithium chloride transformation following the manufacturer's instructions. Zeocin resistant colonies were picked from selective plates and used to inoculate small-scale expression cultures of minimal glycerol medium (1.34% w/v yeast nitrogen base, 1% w/v glycerol, 4 × 10^−5^% biotin w/v) containing histidine (0.004% w/v) and grown in an orbital incubator (250 rpm) at 28–30°C. Cultures were induced when an A_600_ of 10 was reached by replacing the medium with minimal methanol medium containing histidine (0.5% v/v methanol replacing the glycerol) and cultured for 48 hours with a further addition of methanol (0.5% v/v) 24 hours postinduction. Cells were harvested by centrifugation (2500 g, 15 minutes at 4°C), washed with ice cold H_2_O and resuspended in three times the pellet volume of ice cold YeastBuster (Merck) with protease inhibitors, incubated for 90 minutes with agitation and cell debris removed by centrifugation (500 g, 1 minute at 4°C).

### 2.6. Immunoblot Analysis of AUX1 Expression

Cell lysates were quantified by a detergent compatible protein assay (BioRad) and (10 *μ*g) aliquots were resolved on 10% w/v SDS-PAGE gels, electroblotted and recombinant epitope-tagged AUX1 protein (ca. 45–50 kDa) identified by Western blotting with rabbit anti-HA or rabbit anti-FLAG monoclonal antibodies (both from Axxora; 1 : 2000 to 1 : 5000 dilutions) as appropriate. Following removal of the primary antibody, AUX1 expression was determined using horseradish peroxidase conjugated secondary antibody (goat anti-rabbit-HRP, DAKO, 1 : 2000) and enhanced chemiluminescence (SuperSignal West Pico, Pierce).

### 2.7. Confocal Microscopy of AUX1-Expressing Mammalian Cells

HEK293T cells were grown on cover slips (100 000 cells per coverslip in a 35 mm dish) and transfected using PEI as described above. After washing with ice cold PBS cells were fixed and permeabilized in methanol : acetone (1 : 1) for 20 minutes at −20°C and washed again. Nonspecific binding was blocked by incubating in 3% (w/v) BSA, 1 mM CaCl_2_, 1 mM MgCl_2_ for 1 hour. Cells were washed in 0.3% (w/v) BSA, 1 mM CaCl_2_, 1 mM MgCl_2_ and probed with anti-FLAG primary antibody at 1 : 1000 (v/v) then anti-rabbit-GFP (Sigma) secondary antibody at 1 : 200 (v/v). Cells were imaged using a Leica SP2 confocal laser scanning microscope. The green fluorescence of GFP was excited using the 488 nm laser line and the DAPI staining using the 405 nm laser. Optical (z) sections were collected at intervals of 0.25 microns and displayed as maximum intensity projection using the associated LCS software.

### 2.8. Blue Native Gel Polyacrylamide Gel Electrophoresis of AUX1

AUX1 containing membranes were solubilised at a protein concentration of 1 mg/mL for 3 hours on ice in 0.1% *n*-dodecyl-*β*-D-maltoside (Calbiochem) in the presence of 20 mM HEPES-KOH, pH 7.4, 250 mM NaCl, 10% v/v glycerol, 2.5 mM MgCl_2_, 1 mM EDTA, 1 mM 2-mercaptoethanol and protease inhibitors. Insoluble material was removed by centrifugation at 100 000 g for 1 hour at 4°C and 10× sample buffer (0.5% w/v G250 Coomassie brilliant blue, 0.75 M 6-aminocaporic acid, 100 mM Bis-Tris-HCl, pH7) added at a volume ratio of 1 : 10 prior to electrophoresis. The solubilised proteins were analyzed by blue native electrophoresis on 6–16% linear polyacrylamide gradient gels as previously described [11].

### 2.9. Interaction of IAA with AUX1 Containing Membranes

AUX1 containing membranes were isolated by ultracentrifugation following cell lysis, and the interaction of [^3^H]-indole-3-acetic acid was determined as described previously [12]. The ability of auxin analogues to displace auxin binding was determined by incubation with 1 mM of displacing compound [12].

### 2.10. Detergent Solubilisation of HA-AUX1

Detergents were investigated for their ability to extract/solubilize HA-AUX1 from insect cell membrane preparations. AUX1 containing membranes (100 *μ*g at a protein concentration of 1 mg/mL) and detergent (between 3 and 8-fold critical micelle concentration) were combined in solubilization buffer (20 mM MOPS, 200 mM NaCl, 1.5 mM MgCl_2_, 20% (v/v) glycerol, pH 7.4) and incubated at 4°C for 60 minutes with end-over-end mixing. Insoluble material resistant to detergent extraction was pelleted by ultracentrifugation at 100 000 g for 1 hour at 4°C and resuspended in 100 *μ*L 10% SDS (w/v). Equivalent percentages of the solubilised (i.e., in the supernatant following detergent extraction) and insoluble material were analysed by SDS-PAGE and immunoblotting with anti-HA antibodies. Solubilisation in the presence of a strong ionic detergent (SDS) served as a positive control as 100% of the HA-AUX1 is extracted under these conditions.

### 2.11. Transport of IAA into Mammalian Cells

Auxin transport assays [13] were performed in AUX1 expressing HEK293T and U2OS cells. Following transfection cells were incubated at 37°C for 30 minutes in Ringers buffer, (115 mM NaCl, 2.5 mM KCl, 1.8 mM CaCl_2_, 1 mM NaHCO_3_, 10 mM HEPES-NaOH, 1 mM MgCl, pH 6.4), containing 2 *μ*M [^3^H]-IAA. Transport was terminated by several rapid washes with ice cold Ringers buffer containing 5 mM IAA, and the cells were then resuspended in 100 *μ*L buffer, and lysed by addition of SDS to a final concentration of 2.5% w/v added and incubated at 37°C for 30 minutes to allow complete cell lysis. An aliquot was removed for protein assay and Western blot to confirm protein expression and the remainder used to determine radioactive content of the cells using a liquid scintillation counter. Each sample was performed in at least duplicate and corrected values expressed as the rate of transport in fmoles IAA/minute/mg protein. To enable comparison of data from different transfections, activity was normalised such that the accumulation observed in pcDNA_L2_His_6_3XFLAG transfected cells was set to 100%.

## 3. Results and Discussion

We investigated several different nonplant expression systems for their ability to express the AUX1 protein. The heterologous systems selected included mammalian cell lines (HEK293T—an embryonic kidney cell line [7], and U2OS—an osteosarcoma cell line [8]), baculovirus-mediated infection of insect cells, a yeast expression system, and a bacterial expression system. The relative merits of these systems in terms of expression level, reliability of expression, AUX1 protein function (binding or transport), and scalability are discussed below. Our studies involved three epitope-tagged versions of AUX1 to enable identification of expressed protein and also to facilitate longer-term purification strategies. Three differently tagged versions were employed (see Methods), and these are shown schematically in Figure 1. 

**Figure 2:**
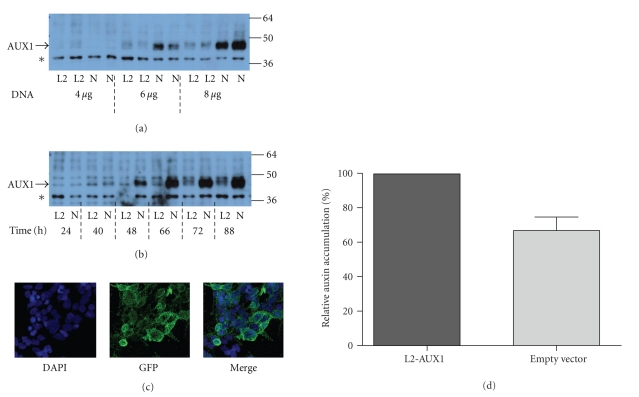
Expression of epitope-tagged AUX1 in mammalian cells. (a) HEK293T cells were transfected using polyethyleneimine with the indicated amounts of recombinant L2-His_6_3xFLAG or N-His_6_3xFLAG epitope-tagged AUX1 DNA (L2 and N, resp., below each lane), and harvested 72 hours posttransfection. (b) HEK293T cells were transfected using 8 *μ*g of either L2-His_6_3xFLAG-AUX1 or N-His_6_3xFLAG-AUX1 epitope-tagged AUX1 DNA and harvested at the indicated times posttransfection. Cells were lysed by sonication, and 10 *μ*g of lysates were resolved by SDS-PAGE and identified by western blotting with anti-FLAG antibodies. The asterisk identifies a nonspecific protein reacting with the anti-FLAG antibody. Molecular weight (kDa) of marker proteins is denoted at the right-hand side of the figure. (c) HEK293T cells were transfected on coverslips in 6-well dishes with cDNA encoding N-His_6_3XFLAG-AUX1 and were visualised 48 hours later by confocal microscopy after immunoblotting with an anti-FLAG primary antibody (1 : 100 dilution) and a GFP-conjugated secondary antibody (1 : 200). Cell nuclei were counter stained with DAPI. (d) Transport of [^3^H]-IAA into U2OS cells transiently transfected with L2-His_6_3XFLAG-AUX1 compared to transport into cells transfected with empty vector. Data is expressed as a percentage of the transport rate into AUX1-transfected cells and represents the mean (± standard error) of 9 independent experiments with 2–4 determinations of transport in each transfection.

Expression in HEK293T cells (Figure 2 ) was dependent on both the epitope tag and its location as well as the quantity of DNA transfected (Figure 2(a)) and the time to harvesting posttransfection (Figure 2(b)). Optimal expression was obtained 88 hours posttransfection with 8 *μ*g of DNA per well, with N-His_6_3XFLAG AUX1 expression being greater than that of L2-His_6_3XFLAG AUX1. Protein was effectively trafficked to the cell membrane as observed with confocal microscopy (Figure 2(c)). Analysis of function of AUX1 was determined using whole-cell radioisotope accumulation assays with [^3^H-IAA], which demonstrated that transfection of vector encoding L2- His_6_3XFLAG AUX1 was capable of causing accumulating of IAA in transfected mammalian cells to a significantly greater degree than empty vector controls (*P* < .01, paired *T*-test, *n* = 9; see Figure 2(d)), although function was tag-position dependent as the N-His_6_3XFLAG construct was not transport competent (data not shown). In spite of these studies, which have been paralleled in the expression and characterisation of other auxin transporters, including the efflux transporters PIN2 and PIN7 and AtABCB1 in HeLa cells [13, 14], the limited ability to scale up the expression capacity precludes attempts to use this system for purification of AUX1. 

**Figure 3:**
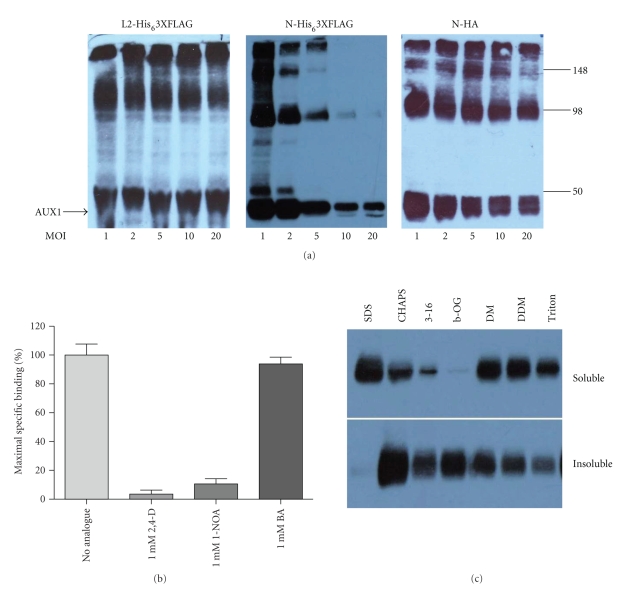
Expression of epitope-tagged AUX1 in insect cells. (a) Sf9 cells at densities of 2 × 10^6^ cells/mL were infected with recombinant baculoviruses expressing epitope-tagged AUX1 at a range of multiplicity of infections (MOIs) and harvested at 24–96 hours postinfection (hpi). Cell lysates (10 *μ*g) were resolved by SDS-PAGE and identified by western blotting with appropriate antibodies directed towards the epitope tags. Panels show data for a 72-hour postinfection only. Molecular weight (kDa) of marker proteins is denoted at the right-hand side of the figure. (b) Auxin binding to AUX1-containing membranes (72 hours postinfection) was assessed by a centrifugation-based radioisotope binding assay [12]. AUX1 displacement could be observed when membranes were incubated with 1 mM auxin analogues such as 2,4-D and 1-NOA but not when the unrelated weak acid benzoic acid (BA) was applied. (c) Solubilisation of HA-AUX1 from insect cell membranes. 100 *μ*g of membranes were incubated for 60 minutes at 4°C with detergents at greater than 2X critical micelle concentration. Solubilised material was separated from insoluble material by ultracentrifugation and equal percentages of the two fractions resolved by SDS-PAGE and immunoblotting. Detergent abbreviations: 3–16: Zwittergent 3–16 (Calbiochem); *β*-OG: *β*-octyl-glucoside; DM: *n*-decyl-*β*-D-maltoside decylmaltoside; DDM: *n*-dodecyl-*β*-D-maltoside.

The baculovirus expression system is well documented for its ability to express high levels of recombinant functional heterologous proteins [15]. The cells can grow in suspension making scale up facile and cost-effective, once recombinant baculoviruses are generated. For optimised expression in insect cells we compared both *Spodoptera frugiperda * Sf9 and *Trichoplusia ni* HighFive cell lines and identified Sf9 as the best line for AUX1 expression (comparison not shown). Maximum expression in Sf9 cells was obtained with infection at a multiplicity of infection (MOI) of 1 (Figure 3(a)), with higher MOIs not producing any increase in protein expression, and subsequent culture for 72 hours posttransfection for all three recombinant viruses. 

All AUX1 constructs expressed in insect cell membranes were functional at least in terms of their interaction with the transport substrate indole-3-acetic acid, and the effective displacement of IAA by auxinic compounds but not by unrelated weak acids (Figure 3(b)). Further details of these initial step in transporter's catalytic cycle have been given recently [12]. Further interpretation of some of these data demonstrates the feasibility of using the insect cell systems for future purification studies of AUX1. Our published maximal binding of IAA to AUX1 is ca. 12 pmoles of IAA/mg membrane protein [12]. Using a demonstrated 1 : 1 stoichiometry [12] this equates to 6 *μ*g AUX1/mg membrane protein. The ease of scaling of insect cell culture enables us to produce 1 g of total membrane protein per litre culture (equivalent to 2 × 10^9^ cells), equivalent to approximately 6 mg of AUX1. Secondly, preliminary solubilisation of AUX1 from insect cell membranes indicates that a range of nonionic detergents including dodecyl-maltoside and decyl-maltoside is able to extract the protein from membranes (Figure 3(c)), underpinning future purification strategies. The disadvantage of the insect cell system is that transport studies are not possible due to viral induced loss of cell integrity 2-3 days after infection. Compromising the expression levels with shorter infection times and reduced MOIs might ameliorate this issue.

**Figure 4:**
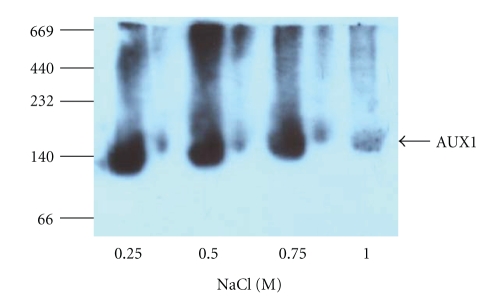
Blue native PAGE analysis of AUX1 expression in insect cells. N-HA-AUX1 membrane fractions from insect (Sf9) cells were solubilised in 0.1% (w/v) DDM in the presence of increasing concentrations of NaCl and were resolved by BN-PAGE on 6–16% gradient gels, transferred to PVDF and identified by immunoblotting with anti-HA antibodies. Molecular weight (kDa) of marker proteins is denoted at the left-hand side of the figure.

In both mammalian and insect cell expression systems we made three comparable observations. Firstly, the expression level of the L2-His_6_-3xFLAG constructs was much lower than that for N-His_6_-3xFLAG AUX1 construct reflecting that the tag position and identity are likely to be critical determinants of the success of a heterologous expression system (lane-by-lane comparison is shown in Figure 2  for mammalian cell expression). Similar data was obtained for insect cell expression of AUX1 (not shown). Secondly, despite a predicted molecular weight of 54 kDa, AUX1 constructs routinely migrated at just less than 50 kDa on SDS-PAGE. Native AUX1 from root cultures also runs with an apparent faster mobility than predicted, a phenomenon not unusual for highly hydrophobic membrane proteins [3]. Thirdly, we observed that HA-AUX1 migrated as multiple bands on SDS-PAGE, with apparent dimerization and higher-order oligomerization, resistant to SDS and reducing agent denaturation (see, e.g., Figure 3). In order to analyse this further (and rule out detergent-induced aggregation of the protein as being responsible), we performed blue native PAGE (BN-PAGE) on HA-AUX1 containing membranes, where separation of the protein and determination of the native molecular weight are driven by the pore size of the gradient acrylamide gel [11]. With this analysis we showed that HA-AUX1 is expressed as a trimer in insect cells with a molecular weight close to 150 kDa (Figure 4). The natural oligomeric state of the protein remains unknown but members of related transport families (i.e., of ammonium) are known to assemble into trimeric species [16].

**Figure 5:**
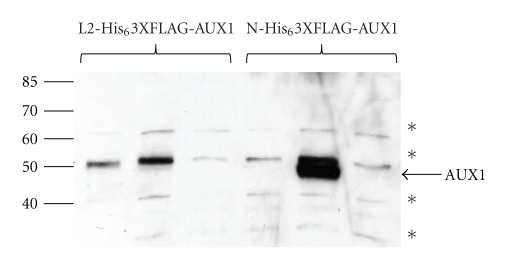
Expression of epitope-tagged AUX1 in *P. pastoris*. *P. pastoris* strain KM71H transformed with plasmids encoding L2-His_6_3xFLAG-AUX1 or N-His_6_3xFLAG-AUX1 was grown, induced, and harvested as described in the Methods. Of the 32 cell lysates analysed by Western blotting (anti-FLAG antibody), only 6 are shown here for clarity, the other 26 showing no AUX1 expression. AUX1 was observed in a single N-His_6_3xFLAG-AUX1 culture (lane 5). A number of nonspecific bands reacting with the anti-FLAG antibody are denoted with asterisks, including one migrating just higher than the AUX1 band.

The methylotrophic yeast *Pichia pastoris* should combine the advantages of a microorganism-based expression system (high yield and facile scale up), whilst also retaining the eukaryotic translation and trafficking machinery associated with membrane protein processing. The commercially available *P. pastoris* expression system takes advantage of this by using the alcohol oxidase promoter, which is methanol induced and tightly regulated, to drive heterologous expression of the protein of interest. It has successfully been used to express a wide range of membrane integrated transport proteins [17, 18]. Two *P. pastoris* strains GS115 and KM71H were transformed with AUX1 containing plasmids, and integration of the transgene was confirmed by PCR analysis (data not shown). However, expression analysis of 16 independent transformants from both strains with N- and L2-His_6_3xFLAG constructs identified only a single strain/construct combination which expressed AUX1 (Figure 5). Due to this unreliability—discussed in more detail in relation to the different promoters in *Pichia* expression systems [18]—we did not attempt to perform any functional analysis of Pichia expressed AUX1. Notwithstanding this, other yeast systems have been used successfully for the expression and characterisation of a number of auxin transporters, notably PIN transporters PIN2 and PIN7, and the ATP binding cassette (ABC) transporter AtABCB1. For *S. cerevisiae * based studies this has required the use of strains deficient in numerous confounding transporters [13, 14]. Most recently, an *S. pombe* expression system has been used which is able to show functional expression of 3 different classes of auxin transporter, namely, AUX-LAX, PIN, and ABCB, and this seems likely to supersede other yeast systems for auxin transporter investigation [19].

The gram positive lactic acid bacterium *Lactococcus lactis* was selected as a candidate for a prokaryote-based expression of AUX1 because of reports on its successful use to express and functionally characterize a range of prokaryotic and eukaryotic membrane transporter proteins (reviewed in [20]). *L. lactis * is the model lactic acid bacterium, and a controlled inducible protein expression system for heterologous proteins has been described [21]. Although the insertion of the AUX1 gene into the pNZ8048 vector was confirmed by DNA sequencing no expression was observed for any of the tagged AUX1 constructs under the range of conditions used, in contrast to a control expression vector for an unrelated ATP binding cassette (ABC) protein. It has previously been shown that the success of expression in the *L. lactis * system is influenced by the N-terminal region of the transport protein [22]. It can therefore be hypothesised that N-terminal 50 amino acids of AUX1 prior to the first predicted TM helix is unsuitable for expression in *L. lactis.* Modifications to these regions (as described by Monné and colleagues for a range of mitochondrial carriers including the ADP/ATP carriers ACC1 and ACC2 [22]) may eventually lead to expression of AUX1 in this *L. lactis*.

## 4. Conclusion

We have examined four systems for their suitability to express the plant hormone transporter AUX1. The position and the nature of the epitope tag are an important consideration, as is the desired outcome, that is, transport studies, purification of protein, and so forth. Of the four systems the prokaryotic *L. lactis* was unsuccessful in our hands, with no expression observed. The methylotrophic yeast *P. pastoris* gave limited and unreliable success. In contrast, transiently transfected mammalian cells and a baculovirus infected insect cell systems enabled us to express AUX1 and assess functionally competence of the protein. These two heterologous systems for AUX1 complement the use of *Xenopus* oocytes and *S. pombe * for studies of auxin transporters [19, 23, 24]. The oocyte system, although technically beyond many research laboratories, has enabled determination of Michaelis-Menten parameters for AUX1 and LAX3. The accessible baculovirus expression system has enabled detailed analysis of binding affinities of auxin transport substrates and inhibitors [12], and this combination of approaches opens the way for similar studies on a range of these proteins. Determination of the biochemistry of auxin importers and exporters will lead to more realistic models of auxin transport, with greater power to predict responses to changes in auxin concentration [25].

## Abbreviations

AUX1: AUXIN RESISTANT 1
ABC: ATP binding cassette
PVDF: Polyvinylidene fluoride
SDS-PAGE: Sodium dodecyl sulfate polyacrylamide gel electrophoresis
BN-PAGE: Blue native polyacrylamide gel electrophoresis
2,4-D: 2,4-dichlorophenoxyacetic acid
1-NOA: 1-naphthylphthalamic acid
BA: Benzoic acid
PBS: Phosphate buffered saline
DMEM: Dulbecco's modified Eagle medium
DM: *n*-decyl-*β*-D-maltoside
DDM: *n*-dodecyl-*β*-D-maltoside.
